# Supplementary material for: Exonuclease Xrn1 regulates TORC1 signaling in response to SAM availability
Source: bioRxiv. 2023 Sep 28:2023.09.28.559955. Preprint. [Version 1] doi: 10.1101/2023.09.28.559955 (PMC10557749; doi:10.1101/2023.09.28.559955)
Supplement: Supplement 3 [file NIHPP2023.09.28.559955v1-supplement-3.pdf]

bioRxiv preprint doi: https://doi.org/10.1101/2023.09.28.559955; this version posted September 28, 2023. The copyright holder for this preprint (which was not certified by peer review) is the author/funder, who has granted bioRxiv a license to display the preprint in perpetuity. It is made available under aCC-BY 4.0 International license.

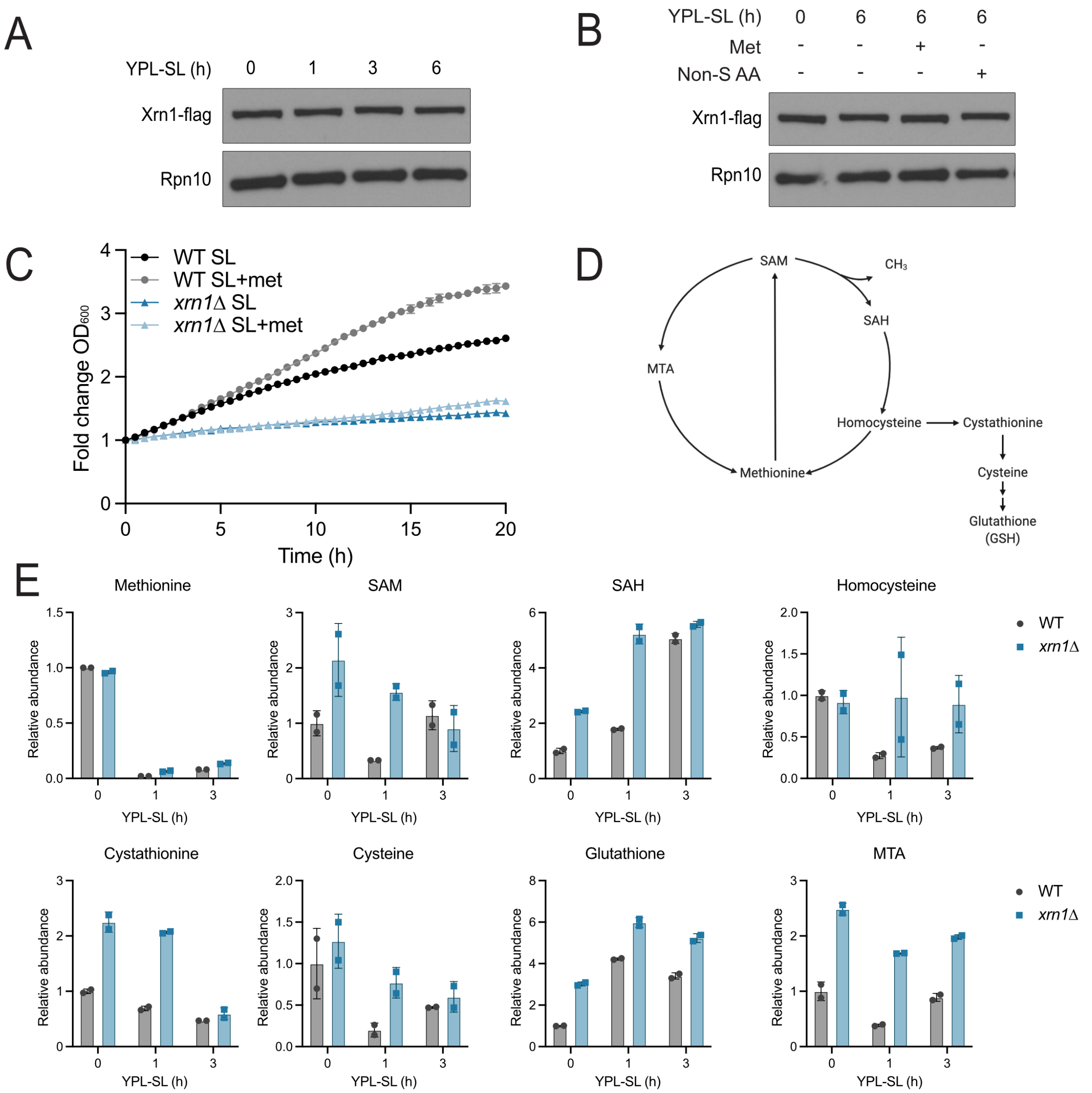

Fig. S1 | Loss of Xrn1 causes elevated levels of SAM and other sulfur-containing metabolites. **(A)**, **(B)** Xrn1 protein abundance is not altered under different metabolic conditions. Anti-flag Western blot assessing protein amounts of Xrn1 under the indicated conditions. **(C)** Methionine restores growth of WT but not *xrn1Δ* cells. Growth curve measuring OD_{600} of WT or *xrn1Δ* cells in the indicated media. OD_{600} was measured every 30 min. Fold change is plotted. The data are represented as mean ± SD (n=3). **(D)** Schematic of sulfur-containing metabolites in yeast produced from methionine and transsulfuration. **(E)** Many sulfur-containing metabolites are elevated in cells lacking Xrn1. WT and *xrn1Δ* cells were grown in the indicated conditions. Metabolite samples were collected and analyzed by LC-MS/MS. Data are represented as mean ± SD (n=2). The metabolite data are also presented in Table S1.

bioRxiv preprint doi: https://doi.org/10.1101/2023.09.28.559955; this version posted September 28, 2023. The copyright holder for this preprint (which was not certified by peer review) is the author/funder, who has granted bioRxiv a license to display the preprint in perpetuity. It is made available under aCC-BY 4.0 International license.

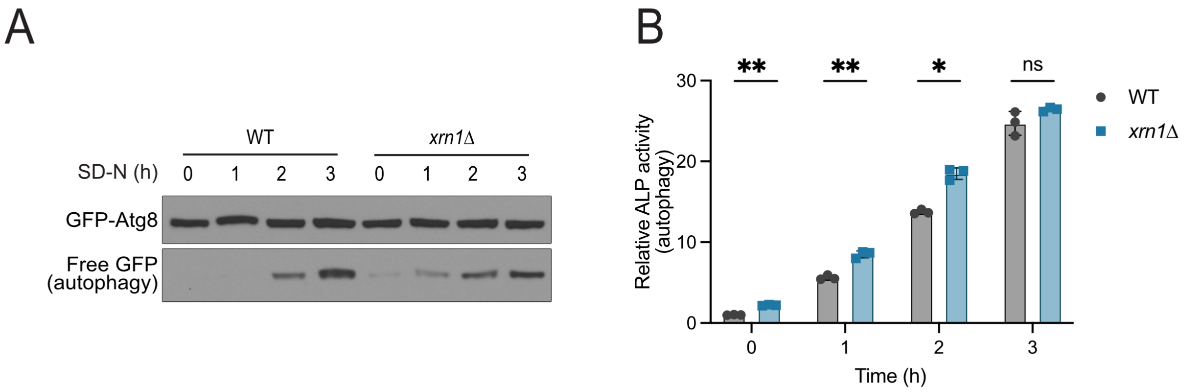

Fig. S2 | Xrn1 is a negative regulator of nitrogen starvation-induced autophagy. **(A)** Autophagy under nitrogen starvation conditions is induced more rapidly in *xrn1*Δ cells. WT and *xrn1*Δ cells harboring a centromeric plasmid expressing GFP-Atg8 were grown to mid-log phase in YPD then starved for nitrogen (SD-N) for the indicated times. Free GFP, indicative of autophagy induction, was detected by Western blot. **(B)** Autophagy under nitrogen starvation conditions in *xrn1*Δ cells as monitored by ALP assay. Cells were grown to mid-log phase in YPD then starved of nitrogen (SD-N) for the indicated times. ALP activity was measured and normalized to the WT cells in rich media. Mean±SD, n=3, statistical analysis performed using student's t-test.

bioRxiv preprint doi: https://doi.org/10.1101/2023.09.28.559955; this version posted September 28, 2023. The copyright holder for this preprint (which was not certified by peer review) is the author/funder, who has granted bioRxiv a license to display the preprint in perpetuity. It is made available under aCC-BY 4.0 International license.

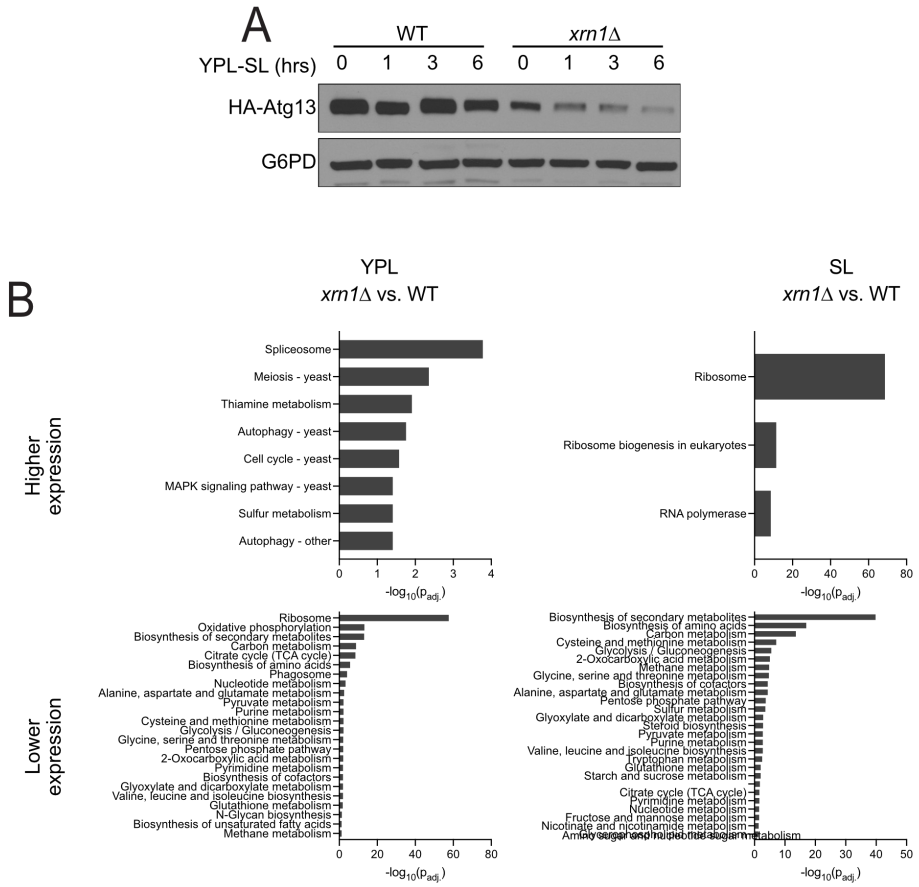

Fig. S3 | Amounts of select autophagy mRNAs and proteins in cells lacking Xrn1.
(A) Key autophagy protein Atg13 exhibits reduced abundance in cells lacking Xrn1. HA-tagged Atg13 protein abundance was assayed in WT and *xrn1*Δ cells following methionine deprivation for the indicated times by Western blotting. **(B)** Gene ontology enrichment from RNA-seq identifies gene groups that are altered in *xrn1*Δ cells.

bioRxiv preprint doi: https://doi.org/10.1101/2023.09.28.559955; this version posted September 28, 2023. The copyright holder for this preprint (which was not certified by peer review) is the author/funder, who has granted bioRxiv a license to display the preprint in perpetuity. It is made available under aCC-BY 4.0 International license.

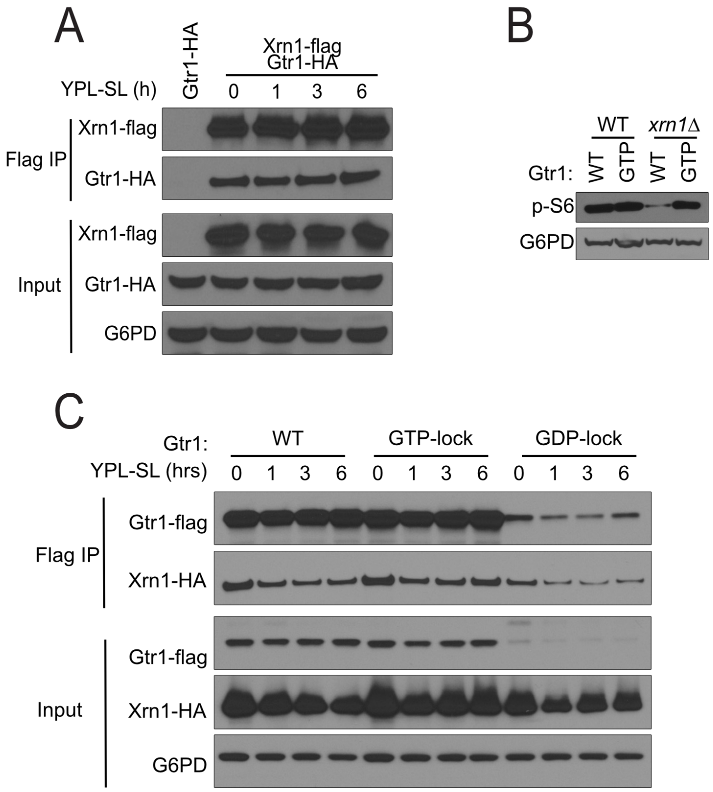

Fig. S4 | Xrn1 interacts with Rag GTPase Gtr1.
(A) Xrn1 and Gtr1 interact independent of methionine availability. Cells expressing flag-tagged Xrn1 and HA-tagged Gtr1 were grown in YPL and switched to SL for the indicated times. Interaction between these proteins was assessed by co-IP followed by Western blot. **(B)** GTP-locked mutation of Gtr1 restores TORC1 activity in cells lacking *xrn1*Δ, assayed by Western blot for phosphorylated S6 ribosomal protein. **(C)** Xrn1 preferentially interacts with the GDP-locked form of Gtr1 by co-IP. Cells expressing flag-tagged Gtr1 constructs and HA-tagged Xrn1 were grown in the indicated conditions. Interaction between these proteins was assessed by co-IP followed by Western blot. Note the GDP-locked (S20L) mutation destabilizes the Gtr1 protein compared to WT or GTP-locked (Q65L) mutation.

bioRxiv preprint doi: https://doi.org/10.1101/2023.09.28.559955; this version posted September 28, 2023. The copyright holder for this preprint (which was not certified by peer review) is the author/funder, who has granted bioRxiv a license to display the preprint in perpetuity. It is made available under aCC-BY 4.0 International license.

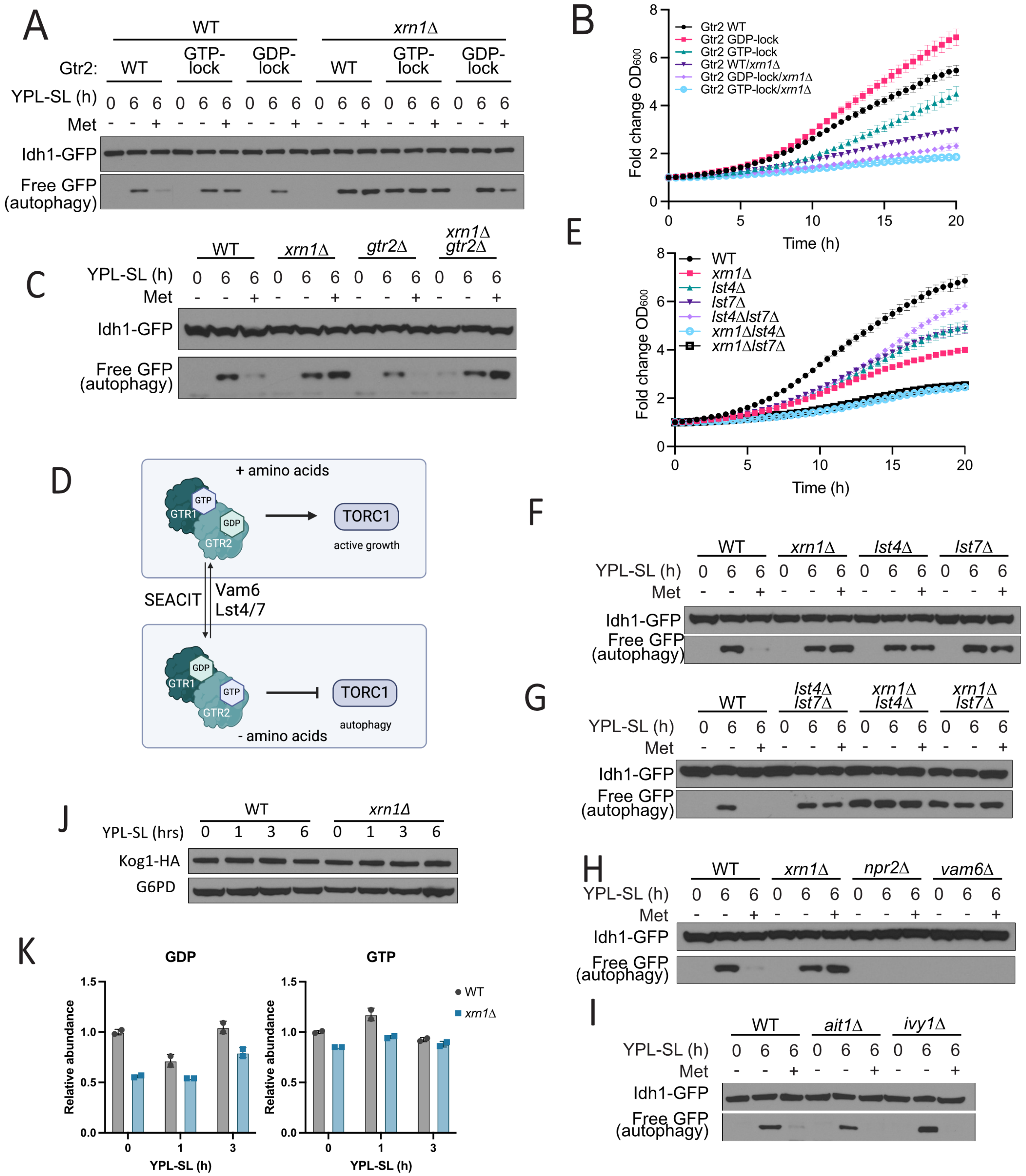

Fig. S5 | Xrn1 does not act through known Gtr1/Gtr2 regulatory proteins.
(A) GTP- and GDP-locked mutations in Gtr2 synergize with loss of Xrn1 in regulating autophagy following methionine deprivation. The indicated strains expressing point mutations of Gtr2 to lock it in its GTP-binding (Q66L) or GDP-binding (S23L) state were grown in YPL and then switched to SL for 6 h, in the absence or presence of 1 mM methionine. Autophagy was measured by the GFP cleavage assay. **(B)** GTP- and GDP-locked mutations in Gtr2 exacerbate the growth defect of *xrn1*Δ cells. The indicated strains were grown in YPL and OD_{600} was measured every 30 min.

bioRxiv preprint doi: https://doi.org/10.1101/2023.09.28.559955; this version posted September 28, 2023. The copyright holder for this preprint (which was not certified by peer review) is the author/funder, who has granted bioRxiv a license to display the preprint in perpetuity. It is made available under aCC-BY 4.0 International license.

Fold change in OD_{600} is plotted. **(C)** Cells lacking both Xrn1 and Gtr2 exhibit enhanced autophagy. The indicated strains were assayed for autophagy as in (A). **(D)** Schematic depicting how the nucleotide binding states of Gtr1 and Gtr2 are controlled by GAPs and GEFs. **(E)** Growth curves of cells lacking Xrn1 and either Lst4 or Lst7 reveals a synthetic growth defect. The indicated strains were grown in YPL and OD_{600} was measured every 30 min. Fold change in OD_{600} is plotted. **(F)** Loss of the Gtr2 GAPs Lst4 and Lst7 phenocopy the methionine-insensitive autophagy phenotype of *xrn1*Δ mutants. Autophagy was assayed as in (A). **(G)** Cells lacking Xrn1 in combination with Lst4 or Lst7 exhibit significantly enhanced autophagy. Autophagy was assayed as described as in (A). **(H)** Loss of Vam6 phenocopies *npr2*Δ cells in regulation of autophagy. Vam6 is annotated as a GEF for Gtr1. The indicated strains were assayed for autophagy as in (A). **(I)** Loss of Ait1 and Ivy1 do not play a role in regulation of autophagy in response to methionine deprivation. The indicated strains were assayed for autophagy as in (A). **(J)** Abundance of Kog1 protein is not altered in *xrn1*Δ compared to WT. Either WT or *xrn1*Δ with HA-tagged Kog1 were grown in the indicated conditions, and Kog1 protein abundance was assayed by Western blotting. **(K)** Targeted metabolomics reveals slightly reduced levels of both GDP and GTP in cells lacking Xrn1. WT and *xrn1*Δ cells were grown in the indicated conditions. Metabolites were extracted at the indicated times and analyzed by targeted LC-MS/MS.

bioRxiv preprint doi: https://doi.org/10.1101/2023.09.28.559955; this version posted September 28, 2023. The copyright holder for this preprint (which was not certified by peer review) is the author/funder, who has granted bioRxiv a license to display the preprint in perpetuity. It is made available under aCC-BY 4.0 International license.

Table S3: Yeast strains used in this study.

All strains are in the prototrophic CEN.PK background.

Genotype	Reference
MAT a, MAT α	[12]
MAT α xrn1Δ::KanMX	This study
MAT a IDH1-GFP::NAT	This study
MAT a IDH1-GFP::NAT, xrn1Δ::KanMX	This study
MAT α XRN1-FLAG::KanMX	This study
MAT a IDH1-GFP::NAT, XRN1-FLAG::KanMX	This study
MAT a pho8:TEF1^{P}-pho8Δ60::KanMX, pho13Δ::NAT	[1]
MAT a pho8:TEF1^{P}-pho8Δ60::KanMX, pho13Δ::NAT, xrn1Δ::HYG	This study
MAT α IDH1-GFP::NAT, npr2Δ::HYG	[14]
MAT α IDH1-GFP::NatMX, xrn1Δ::KanMX, npr2Δ::HYG	This study
MAT α XRN1-FLAG::KanMX, NPR2-HA::HYG	This study
MAT α NPR2-HA::KanMX	This study
MAT α XRN1-FLAG::KanMX, GTR1-HA::NAT	This study
MAT α GTR1-HA::NAT	This study
MAT a IDH1-GFP::HYG, gtr1Δ::KanMX	This study
MAT α IDH1-GFP::HYG, gtr1Δ::KanMX, xrn1Δ::NAT	This study
MAT a IDH1-GFP::HYG, GTR1-FLAG::KanMX	This study
MAT α IDH1-GFP::HYG, GTR1(Q65L)-FLAG::KanMX	This study
MAT α IDH1-GFP::HYG, GTR1(S20L)-FLAG::KanMX	This study
MAT a IDH1-GFP::HYG, GTR1-FLAG::KanMX, xrn1Δ::NAT	This study
MAT α IDH1-GFP::HYG, GTR1(Q65L)-FLAG::KanMX, xrn1Δ::NAT	This study
MAT α IDH1-GFP::HYG, GTR1(S20L)-FLAG::KanMX, xrn1Δ::NAT	This study
MAT α GTR1-FLAG::HYG, NPR2-HA::KanMX	This study
MAT α GTR1-FLAG::HYG, NPR2-HA::KanMX, xrn1Δ::NAT	This study
MAT α GTR1-FLAG::HYG, GTR2-HA::KanMX	This study
MAT α GTR1-FLAG::HYG, GTR2-HA::KanMX, xrn1Δ::NAT	This study

bioRxiv preprint doi: https://doi.org/10.1101/2023.09.28.559955; this version posted September 28, 2023. The copyright holder for this preprint (which was not certified by peer review) is the author/funder, who has granted bioRxiv a license to display the preprint in perpetuity. It is made available under aCC-BY 4.0 International license.

MAT α GTR1-FLAG::KanMX, XRN1-HA::HYG	This study
MAT α GTR1 (Q65L)-FLAG::KanMX, XRN1-HA::HYG	This study
MAT a GTR1 (S20L)-FLAG::KanMX, XRN1-HA::HYG	This study
MAT α GTR1-flag::HYG, KOG1-HA::KanMX	This study
MAT α GTR1-flag::HYG, KOG1-HA::KanMX, xrn1Δ::NAT	This study
MAT a KOG1-HA::KanMX	This study
MAT a IDH1-GFP::KanMX, GTR2-FLAG::HYG	This study
MAT a IDH1-GFP::KanMX, GTR2 (S23L)-FLAG::HYG	This study
MAT a IDH1-GFP::KanMX, GTR2 (Q66L)-FLAG::HYG	This study
MAT a IDH1-GFP::KanMX, GTR2-FLAG::HYG, xrn1Δ::NAT	This study
MAT a IDH1-GFP::KanMX, GTR2 (S23L)-FLAG::HYG, xrn1Δ::NAT	This study
MAT a IDH1-GFP::KanMX, GTR2 (Q66L)-FLAG::HYG, xrn1Δ::NAT	This study
MAT a IDH1-GFP::NAT, ait1Δ::KanMX	This study
MAT a IDH1-GFP::NAT, ivy1Δ::KanMX	This study
MAT a IDH1-GFP::NAT, lst4Δ::KanMX	This study
MAT a IDH1-GFP::NAT, lst7Δ::KanMX	This study
MAT a IDH1-GFP::NAT, lst4Δ::KanMX, lst7Δ::HYG	This study
MAT a IDH1-GFP::NAT, xrn1Δ::KanMX, lst4Δ::HYG	This study
MAT α IDH1-GFP::NAT, xrn1Δ::KanMX, lst7Δ::HYG	This study
MAT a IDH1-GFP::NAT, gtr2Δ::HYG	This study
MAT a IDH1-GFP::NAT, gtr2Δ::HYG, xrn1Δ::KanMX	This study
MAT α HA-ATG13::NAT	[1]
MAT α HA-ATG13::NAT, xrn1Δ::KanMX	This study
